# Editorial

**Published:** 1875-02

**Authors:** 


### Editorial.

### PERSONAL.

It is with much gratification that we learn, that Dr. C. W. Spalding, of St. Louis, has, after an absence of about seven years from the ranks of the dental profession, again entered upon the practice. The Dr. has undoubtedly all the while felt the attraction to his first love, and it became so strong that on the first day of 1875, he was drawn in.

We know that the Dr. will be most heartily welcomed into the profession again. As an honorable, high-minded and most successful practitioner he was known throughout this country, and indeed throughout the world where dentistry is known. Long may he occupy his present position, and never again forsake his first love.

### OHIO DENTAL COLLEGE ASSOCIATION.

The annual meeting of the Stockholders of the Ohio Dental College, and others interested, will be held in the College on Tuesday, March 2d, at 2 o'clock, p. m.

It is desirable that there be a full and prompt attendance, so that the business may be at once dispatched. There is important work for the body to do at every annual meeting. Now, gentlemen, be on hand at the hour.

### COMMENCEMENT.

The Commencement Exercises of the thirtieth annual session of the Ohio Dental College will be held in the College, in this city, on Thursday, March 4th, at 7 1-2 o'clock, p. m. The annual address will be delivered by Dr. Joseph Richardson, of Terre Haute, Ind., and formerly Prof. of Mechanical Dentistry, in this Institution. A cordial invitation is extended to all interested to be present.

### MISSISSIPPI VALLEY DENTAL SOCIETY.

The thirty-first annual meeting of this society will be held in the lecture room of the dental college in this city, beginning Wednesday, March 3d, at 10 o'clock, a. m. We hope to meet a large number of the profession on that occasion. This is the oldest dental society in the world, but it is more vigorous than in its youth; and is more efficient than ever before.

The Executive Committee have prepared a good programme for papers and discussions. There will most probably be quite a number of good papers on the various subjects. We append the subjects selected, which we feel assured will be a strong inducement to all within reach, at least, to be present.

SUBJECTS FOR REVIEW AND DISCUSSION BEFORE THE MISSISSIPPI VALLEY DENTAL SOCIETY, MARCH, 3d, 1875.

1st. Is it good practice to extract teeth from a crowded denture to prevent or arrest decay upon the proximate surfaces of the teeth ?

2d. Irregularities of the teeth; description of cases; methods of treatment.

3d. Sensitive dentine; treatment; topical applications, their relative efficiency and mode of action.

4th Dental hygiene; food in its relation to the development of good teeth.

5th. Dental education.

6th. The relative position of operative and mechanical dentistry.

7th. Filling teeth ; comparative value of different materials; description of methods of operating.

8th. Treatment of dental pulps for their preservation.

9th. Mechanical dentistry; the relative merits of celluloid and rubber as a base for artificial teeth.

Upon several of these subjects it is probable that essays will be written. We hope the members will all come prepared to contribute something of interest to the meeting. By a little *thought* and *preparation*, the usefulness of all our societies may be much increased.

### BIBLIOGRAPHICAL.

Below we give the notice of a forthcoming work that will doubtless be of considerable interest to many in the profession. It is being prepared under the auspices of the Michigan Dental Protective Union, by the Hon. C. C. Burt, of Jackson, Mich. There is an immense amount of material for such a work, and the skill and labor will be displayed more in rejecting than in appropriating.

Let the pith and kernel be retained and the hull and chaff thrown away.

It would be well, in such a work, to have included the conclusion of the contest, if it is ever to be concluded.

The dentists in Michigan have taken up this contest in earnest, and if they go on as they have started they will certainly make a point somewhere. They feel deeply aggrieved by the course pursued by the Court in the case recently tried in Detroit, and their grievances doubtless have a foundation somewhere.

We should be glad to see the profession of Michigan unanimously decide to discard forever rubber for dental purposes, and be done with the intolerable nuisance. They would thereby secure the admiration and thanks of the entire profession, and many others would doubtless immediately follow. They are all bound together in their "Protective Union" and have the power to do or not to do.

"History of the Dental Litigation in America, With the records of the Wetherbee, Newbrough, Gardner, and other cases, as presented in the Smith case, the Root case, and the celebrated Willis' Michigan case, together with briefs, pleadings, proofs, arguments of counsel, opinions thereon of the several Courts of the United States sitting in equity, as well as the several criticisms by Dental and other Journals, containing a short but full history of the production, importation and valuable uses of Rubber, as well as a certified copy of all patents issued thereon, or growing out of its use, in dental business in England, Brussels, France, and America, with copious extracts from dental and scientific journals during the last thirty years, with forms and directions for compounding and molding all kinds of dentures cast or formed in a matrix, with much other valuable information for everybody. Published under the auspices of the Michigan Dental Protective Union. Written and compiled by Calvin C. Burt, A. M., Counselor at Law."

Marriеd—By Rev. W. G. March, Dec. 31st, 1874, at the residence of Mr. Sam'l Woods, of Union Township, Union Co., Ohio, Dr. Henry W. Morey, Marysville, Ohio, and Miss Clara A. Woods.

### "WHAT ABOUT THE RUBBER BUSINESS NOW?"

This question comes to us so often, and so earnestly, that we should like to answer it if we could; but really we don't know much about it, and should care a great deal less, were it not that some of our professional brethren are in such deep tribulation about it, that our sympathetic nature, rather leads us to help them weep, but it is really more through sympathy than grief. We never see a fellow being in real trouble, that the heart does not go out to him and desire to help him.

In the present status of affairs the Rubber Co. seem to have it all their own way, at least so far as decisions in the courts are concerned; but there seems to be some dissatisfaction, because the district courts hang together; but they are compelled to do it, so says Judge Emmons. "Jist so Judge."

The Rubber Co. have their agents on the wing everywhere, gathering or attempting to gather what they regard as the fruits of a great victory. But such success as they desire is not attending their efforts. A great many in the profession are refusing to take license; and many who have till now used it are abandoning its use; and all are seeking a substitute, and the present indications are, that in Celluloid it is found. This material is having a rapid introduction; nearly all the better class of practitioners who are doing much in artificial dentures are testing it, and the report from nearly everyone, thus far, is, that it is as good if not better than rubber. Our observation and experience seem to confirm the latter view. We have examined many cases in which it has been worn from two months to three years, and have yet to find a case in which the mucous membrane is irritated by the presence of the plate, unless there was undue pressure at some point. In the use of rubber plates, there is, in the majority of cases, more or less irritation of the mucous membrane, and in many instances to a marked degree; and that too without undue pressure. As to the cause of this irritation there is a diversity of opinion, but the fact is plain to every close observer. Upon this we may give an opinion at another time.

To any who acquaint themselves with the present method, Celluloid is as easily wrought as rubber. If Celluloid is found, upon proper trial, to be no better than rubber for dental plates, we shall condemn it just as strongly.

There are some important views of this subject that have been kept too much in the back-ground. The great blessing conferred upon mankind by the introduction of rubber for artificial dentures has been largely expatiated upon by some in the profession, such persons usually close their eyes to any other view of the subject, and usually content themselves with the assertion that. "It is a great blessing." If we were going to examine the subject, we might ask; to whom is it a blessing? Is it so to the thousands and tens of thousands of people in this country who have been induced, by the feature of cheap dentures, urged by quacks and scoundrels, to sacrifice millions of priceless teeth that would with proper care have performed their functions during life?

Is it a blessing to those who, in view of the cheapness of artificial dentures, neglect their natural teeth till they are injured, diseased and ruined? Is it a blessing to those who, from ruthless extraction of the natural teeth, have the face and features irreparably marred and distorted for life? Is it a blessing to those, the beauty, harmony and attractiveness of whose speech is impaired beyond recovery? Is such a loss in any sense compensated for by the possession of a *new beautiful* ten dollar set of artificial teeth on rubber? Is it a blessing to those who, having a partial set of teeth on rubber, jeopardize by its bad influence the health and preservation of a number of good, healthy and useful natural teeth? This has occurred and is occurring in multitudes of cases. Partial dentures on any material, without great care are injurious to good natural teeth; but those constructed of material, the tendency of which is to produce a diseased condition of the mucous membrane and a vitiation of the oral secretions, are doubly so.

Is it a blessing to the dental profession to have a host of incompetent pretenders thrust into its ranks, whose influence, so far as they have any, is to drag the entire profession to their own low degraded level? We have often heard the very best and most efficient in the profession classed with just such as we have here referred to.

Is it gratifying to the student, who with an honorable purpose of devoting from three to five years of his best energy and effort to prepare himself for the practice of an honorable and useful profession, to learn that others are sent out with from four to six week's instruction, who will assume to do all that he ever expects to be able to accomplish? Is it a blessing to the student, who enters upon his course of pupilage with the honest intent of doing all that is necessary, to find that after having spent two or three years, and is pronounced complete by his preceptor, he knows nothing of dental prosthesis except the method of constructing artificial dentures upon rubber? Nine-tenths of all who have entered the practice of dentistry within the last twelve years, know nothing of the methods of making artificial denture with anything except rubber. Many dentists, and some who have been in practice for quite a number of years have told us that they never even saw a denture made on metal plate.

A far larger relative proportion of quacks and incompetents have invaded the dental profession during the last twelve years than ever before; and this too during a time when far greater effort has been exercised by the profession for its education and elevation than before.

Now we do not charge all the quackery in the profession upon the introduction of rubber as a base for artificial teeth; but a large share of it is in existence because it is possible to take any boy or young man of ordinary capacity, and sometimes the veriest dunces are selected, and teach him in four weeks to make a set of teeth on rubber, that will compare with the slop-shop work that is done in one-half of the offices in the country. (And also to stuff teeth with amalgam).

Now this opportunity did not exist before the introduction of rubber. Though there were some who started out on a very short term of pupilage, but it was utterly impossible for them to attain anything like the success that this class now do, and they did not exhibit the audacity that now attaches to the self-constituted dentist.

In those days the principal stock in trade of the empirics was amalgam with the addition of a moderate amount of assumption. The quackery of those days was by no means as intrusive and dangerous as that, that prevails now, which has for its corner-stone and cap-stone too—*Rubber Dentistry.*

---

### THE DENTAL PAIN OBTUNDER.

We are often asked, "How about *Dental Pain Obtunder* now?" And in answer will say, that it does all that has been claimed for it, and even more. It is by far the most efficient agent for reducing hypersensitivness of the dentine that we have used. In our practice, in a large majority of cases, it relieves the sensitiveness entirely; in a few cases it does not produce a complete relief; but in all cases it so far relieves as to make the operation of filling admissible, and that too without drawing unduly upon the patient's endurance.

A few who have tried it, have said, that they find some cases in which it seemed to give no relief, and in others only partial, while in some its action was entirely satisfactory. Now there may be, and probably are, cases, that will not yield to this agent; indeed it would be remarkable if there were not. This may be the result of peculiar susceptibilities, or idiosyncrasies; or the peculiarities of some localities, climate or material, may modify its action. Or the method of application may have much to do, in some instances, with its efficiency. And here we will state that to give it proper facility for action, it should not be diluted, neither before it is put into the cavity nor afterward, and in order to secure the best results the cavity in which it is to be applied, should be protected from moisture, and made absolutely dry, this of course, can only be done, in most cases, with the rubber dam; this being done, moisten a lock of cotton or bibulous paper with the *Obtunder*, and moisten the cavity throughout with it; usually the sensitiveness will pass away instantly, in other cases it will be more gradual, and occasionally it will be so tardy in producing the desired result, that two or more applications will be required. There is probably nothing to be gained by sealing it up in a cavity and keeping it there for any considerable time.

Our satisfaction in its use has been very great indeed; the success thus far with it, for this purpose, is beyond anything we had ever ventured to hope for, in the direction of therapeutic agents.

The questions will naturally arise in the minds of some, "What will be its ultimate effect?" "Does it produce, or is it liable to produce, devitalization of the structure to which it is applied?"

Our observation thus far, leads us to the conclusion that it does not produce, in any case, devitalization of the dentine. In most cases that have been examined after filling, some sensitiveness is present, as is indicated by thermal changes through the filling.

In some cases there is no apparent return of the sensitiveness, at least by the test just mentioned, and the presumption is that the tissue assumes a normal condition. In no instance have we seen anything that would indicate devitalization by the influence of this agent.

The value of this agent may extend further, in dental practice, than in the particular just referred to. We have used it in a number of cases in the management of exposed pulp, with results that we have never attained with any other agent; about this, however, we will say something more when we have further experience.

For the past two months we have used it in nearly all cases of extraction, with apparently decided benefit. The method of applying for this purpose is simply with a napkin or bibulous paper wipe the saliva and mucus from the gum about the tooth, and moisten the surface with the Obtunder; the effect is instantaneous.

Well, we will reserve the remainder of our knowledge of the Obtunder till another time.

